# The role of HCO_3_^–^ in propionate-induced anion secretion across rat caecal epithelium

**DOI:** 10.1007/s00424-021-02565-8

**Published:** 2021-04-29

**Authors:** Jasmin Ballout, Martin Diener

**Affiliations:** Institute for Veterinary Physiology and Biochemistry, Justus Liebig University Giessen, Frankfurter Str. 100, 35392 Giessen, Germany

**Keywords:** Anion secretion, Bicarbonate transport, Caecum, Epithelium, Rat, Short-chain fatty acids

## Abstract

Propionate, a metabolite from the microbial fermentation of carbohydrates, evokes a release of epithelial acetylcholine in rat caecum resulting in an increase of short-circuit current (*I*_sc_) in Ussing chamber experiments. The present experiments were performed in order to characterize the ionic mechanisms underlying this response which has been thought to be due to Cl^−^ secretion. As there are regional differences within the caecal epithelium, the experiments were conducted at oral and aboral rat corpus caeci. In both caecal segments, the propionate-induced *I*_sc_ (*I*_Prop_) was inhibited by > 85%, when the experiments were performed either in nominally Cl^−^- or nominally HCO_3_^−^-free buffer. In the case of Cl^−^, the dependency was restricted to the presence of Cl^−^ in the serosal bath. Bumetanide, a blocker of the Na^+^-K^+^-2Cl^−^-cotransporter, only numerically reduced *I*_Prop_ suggesting that a large part of this current must be carried by an ion other than Cl^−^. In the aboral caecum, *I*_Prop_ was significantly inhibited by mucosally administered stilbene derivatives (SITS, DIDS, DNDS), which block anion exchangers. Serosal Na^+^-free buffer reduced I_Prop_ significantly in the oral (and numerically also in aboral) corpus caeci. RT-PCR experiments revealed the expression of several forms of Na^+^-dependent HCO_3_^−^-cotransporters in caecum, which might underlie the observed Na^+^ dependency. These results suggest that propionate sensing in caecum is coupled to HCO_3_^–^ secretion, which functionally would stabilize luminal pH when the microbial fermentation leads to an increase in the concentration of short-chain fatty acids in the caecal lumen.

**Supplementary Information:**

The online version contains supplementary material available at 10.1007/s00424-021-02565-8.

## Introduction

The caecum, forming a blind sac interconnected between the ileum and the colon, is the largest fermentation chamber of herbivorous or omnivorous non-ruminant animals. Its main function is the metabolic production of short-chain fatty acids such as acetate, propionate, and butyrate, by microbiota living in symbiosis with the mammalian host [20]. Its pendant in ruminant species is the forestomach system, in which large amounts of short-chain fatty acids are produced and absorbed by the epithelium serving as an energy source for the host, i.e., the mammal.

Short-chain fatty acids, the end product of the bacterial carbohydrate metabolism, fulfill multiple functions in the intestinal epithelium. Butyrate, for example, is the preferred energy source for the colonic epithelium [25] and short-chain fatty acids affect epithelial proliferation and differentiation [18]. These bacterial metabolites are recognized by the intestinal epithelium via G-protein coupled receptors, of which several types, i.e., FFAR2 (formerly called GPR43) and FFAR3 (formerly called GPR41), are known [9]. Propionate, especially, seems to be sensed permanently by the epithelium of the large intestine. Originally observed in rat colon, luminal propionate stimulates epithelial FFARs and evokes the release of non-neuronal acetylcholine, i.e., acetylcholine produced and released by the epithelium itself [32, 33]. The functional consequence is the induction of anion secretion by paracrine stimulation of epithelial cholinergic receptors, which can be measured as an increase in short-circuit current (*I*_sc_) in Ussing chamber experiments. A similar response has been observed in rat caecum [6]. Acetylcholine-induced *I*_sc_ has been attributed to Cl^−^ secretion based on partial sensitivity to the Na^+^-K^+^-2Cl^–^-cotransport blocker, bumetanide, in rat colon [32, 33] or strong dependence on the presence of Cl^−^ anions in the caecum [6].

The question arises concerning the physiological significance of anion secretion induced by short-chain fatty acid sensing. In ruminants, an increase in the production of short-chain fatty acids (induced experimentally by intraruminal application of highly digestible carbohydrates) in the forestomach system increases salivary secretion [28]. This HCO_3_^−^-rich fluid serves as a buffer necessary to prevent an acidification of the lumen of the forestomach, which would disturb the complex microbial ecosystem in this fermentation chamber finally leading to a potentially lethal disease, i.e., ruminal acidosis [4]. As many Cl^−^ secreting pathways such as the dominant anion channel in the apical membrane of intestinal epithelial cells, the CFTR (cystic fibrosis transmembrane regulator) channel, are also permeable for HCO_3_^−^ [24], it seems to be of interest to study whether HCO_3_^−^ contributes to the electrogenic response evoked by luminal propionate in the caecum. As rat caecum exhibits large segmental differences in basal ion transport and in the epithelial expression of choline acetyltransferase (ChAT), the key enzyme for the production of acetylcholine [6], we tried to find out in the present study if HCO_3_^−^ transport contributes to propionate-induced anion secretion in rat oral and aboral corpus caeci.

## Material and methods

### Animals

Female and male Wistar rats with a body mass of 180–350 g were used. The animals were bred and housed at the Institute for Veterinary Physiology and Biochemistry of the Justus Liebig University Giessen at an ambient temperature of 22.5 °C and air humidity of 50–55% on a 12-h:12-h light–dark cycle with free access to water and food until the time of the experiment. Animals were killed in CO_2_ narcosis by cervical dislocation followed by exsanguination. Experiments were approved by the named animal welfare officers of the Justus Liebig University (administrative number 577_M) and performed according to the German and European animal welfare law.

### Solutions

The Ussing chamber experiments were carried out in a bathing solution containing (mmol·l^−1^): 107 NaCl, 4.5 KCl, 25 NaHCO_3_, 1.8 Na_2_HPO_4_, 0.2 NaH_2_PO_4_, 1.25 CaCl_2_, 1 MgSO_4_, 12.2 glucose. This solution was gassed with carbogen (5% CO_2_ and 95% O_2_, v/v), tempered at 37 °C and had a pH of 7.4. In the Cl^−^-free buffer, NaCl and KCl were equimolarly replaced by Na gluconate (NaGluc) and K gluconate (KGluc), respectively; CaCl_2_ was replaced by Ca gluconate (CaGluc) in a concentration of 5.75 mmol·l^−1^ (in order to compensate the Ca^2+^-buffering properties of gluconate) [16]. In the Na^+^-free buffer, NaCl was equimolarly replaced by N-methyl-d-glucamine chloride (NMDG Cl). The HCO_3_^−^-free buffer (gassed with 100% O_2_) consisted of (mmol·l^−1^): 140 NaCl, 5.4 KCl, 1.25 CaCl_2_, 1 MgSO_4_, 10 HEPES (N-(2-hydroxyethyl) piperazine-Nʹ-2-ethanesulfonic acid), 12.2 glucose, and had a pH of 7.4.

In several experiments with apically permeabilized epithelia, a K^+^ gradient was applied from the mucosal to the serosal side by increasing the KCl concentration in the standard HCO_3_^−^-buffered solution to 13.5 mmol·l^−1^ in the mucosal compartment, while reducing the NaCl concentration to 98 mmol·l^−1^ in order to maintain isoosmolarity.

### Tissue preparation

The caecum was dissected from the ileum and the proximal colon close to their respective junctions with the ampulla caeci and dislocated from the abdominal cavity. After removal of the mesenterial fat and opening of the apex region, a plastic rod (diameter 5 mm) was introduced. The sac-like caecum was cut open with a scalpel along its minor curvature. The mucosal surface was washed by flushing with an ice-cold bathing solution as described previously [6]. Two rectangular segments, an oral one and an aboral one, were cut out with a scalpel from the corpus caeci for Ussing chamber experiments.

### Ussing chamber experiments

The tissue was fixed in a modified Ussing chamber and bathed with a volume of 3.5 ml on each side of the tissue and a measuring area of 1 cm^2^. The caecal segments were incubated at 37 °C and short-circuited by a computer-controlled voltage-clamp device (Ingenieur Büro für Mess- und Datentechnik Mussler, Aachen, Germany) with correction for solution resistance. Tissue conductance (*G*_t_) was measured every minute by the voltage deviation induced by a current pulse (± 50 μA, duration 200 ms) under open-circuit conditions as described previously [6]. Short-circuit current (*I*_sc_) is expressed as μEq·h^−1^·cm^−2^, i.e., the flux of a monovalent ion per time and area, with 1 μEq·h^−1^·cm^−2^ = 26.9 μA·cm^−2^. A positive *I*_sc_ reflects the secretion of anions (or the electrogenic absorption of cations).

At the start of each experiment, the mucosal and the serosal compartment were washed three times in 5 min intervals with about 15–20 ml fresh buffer (i.e., 5 times the chamber volume) to avoid desensitization of the tissue against endogenous propionate present in the caecal lumen [6]. In the corresponding figures, the maximal increase in *I*_sc_ evoked by mucosal administration of propionate or other drugs is given as a difference to the baseline just prior to administration (∆*I*_sc_).

Baseline parameters of *I*_sc_ and *G*_t_ were calculated by averaging the respective parameter over a period of 3 min. At the end of each experiment, the cAMP-dependent secretagogue forskolin and subsequently, the Ca^2+^-dependent secretagogue carbachol was administered as viability control. These control drugs were administered without a washing step, i.e., in the continuous presence of propionate and putative antagonists.

In those experiments, where the *I*_sc_ did not stabilize, i.e., when drugs were administered during the decaying phase of the nystatin-induced *I*_sc_, the theoretical course of *I*_sc_ was calculated by linear regression analysis as described previously [27]. To do so, the *I*_sc_ 3 min prior administration of the drug (30 data points, as *I*_sc_ was registered every 6 s) was used to calculate the regression line. This regression served to extrapolate the decay of *I*_sc_ in the absence of propionate, which was subtracted from the maximal increase in *I*_sc_ evoked by propionate during the first 10 min after administration of the short-chain fatty acid. For statistical comparisons Table 7, this calculated ∆*I*_sc_ was compared with the change in *I*_sc_ in time-dependent control experiments over the same time interval.

### RT-PCR

For RT-PCR studies, samples from oral and aboral caecum or kidney were transferred into lysis buffer (Macherey–Nagel, Düren, Germany) and homogenized using a mixer mill (NM301; Retsch, Haan, Germany) with a frequency of 30 Hz for about 2 min. Total RNA was extracted using the Nucleo Spin® RNA Plus kit (Macherey–Nagel). RNA was reverse transcribed with Tetro cDNA Synthesis Kit (Bioline, Luckenwalde, Germany).

For the PCR reaction, Bioline®Mangomix (Bioline, Germany) was used with 5 mmol· l^−1^ MgCl_2_. Primers (for sequences and references, see Table 1) were obtained from Eurofins MWG Synthesis, Ebersberg, Germany. Each PCR started with a denaturation period of 0.5 min at 95 °C, followed by an annealing phase of 1 min at 60 °C and an elongation phase of 1 min at 72 °C; the whole cycle was repeated 35 times. For control of the PCR reaction, glyceraldehyde-3-phosphate dehydrogenase (GAPDH) was used. The reaction product was visualized after electrophoresis in a 3% (w/v) high-resolution agarose gel (Carl Roth, Karlsruhe, Germany) and staining with Roti®-Gel Stain (Carl Roth). At least three different biological replicates and two technical replicates were performed for each target gene in the RT-PCR.

**Table 1:** Primers

Target	Gene number	Forward	Backward	Reference
NBCe1A	AF027362.1	5ʹ-GCACAGAGA-GAGGAGGCTT-3ʹ	5ʹ-TGTCTTCCCA-ATGCTAGCCAG-3ʹ	Barmeyer et al. [8]
NBCe1B	AF210250.1	5ʹ-ACTGGAGGAG-CGACGGAAG-3ʹ	5ʹ-TGTCTTCCCA-ATGTCAGCCAG-3ʹ	Barmeyer et al. [8]
NBCe2A	NM212512-1	5ʹ-CTCGGCTGTA-TCACCAACGC-3ʹ	5ʹ-ATTCACTGTG-TCAGGGGCGA-3ʹ	Nejsum et al. [19]
NBCe2B	NM212512.1	5ʹ-ATGGAGAGCT-TCCTGGGCAC-3ʹ	5ʹ-CTCAGCAGAG-ACCAGTCCAG-3ʹ	Nejsum et al. [19]
NBCn1	AF080106.1	5ʹ-ATCTACCTCC-GCTATGTCC-3ʹ	5ʹ-ACTCACAGGC-TTTTCAGGGC-3ʹ	Barmeyer et al. [8]
GAPDH	BC059110	5ʹ-CTACAGCAAC-AGGGTGGTGG-3ʹ	5ʹ-CCACCACCCT-GT TGC TGT AG-3ʹ	Pouokam et al. [23]

Gene numbers refer to http://www.ncbi.nlm.nih.gov. For expected product sizes, see Fig. 7

### Drugs

Bumetanide, 1-EBIO (1-ethyl-2-benzimidazolinone; Tocris, Bristol, UK), and forskolin were dissolved in ethanol (final maximal ethanol concentration 0.25% (v/v)). DIDS (4,4ʹ-diisothiocyanato-stilbene-2,2ʹ-disulfonic acid disodium salt) and methazolamide were dissolved in dimethylsulfoxide (DMSO; final maximal DMSO concentration 0.2% (v/v)). Carbachol, DNDS (4,4ʹ-dinitrostilbene-2,2ʹ-disulfonic acid disodium salt), SITS (4-acetamido-4ʹ-isothiocyanato-stilbene-2,2ʹ-disulfonic acid sodium salt), and sodium propionate were dissolved in aqueous stock solutions. Nystatin was dissolved in DMSO (final DMSO concentration 0.2% (v/v)); the stock solution was ultrasonified immediately before use. If not indicated differently, drugs were from Sigma, Taufkirchen, Germany.

### Statistics

Results are given as mean ± standard error of the mean (SEM) with the number (*n*) of investigated tissues. For the comparison of two groups either Student’s *t*-test (paired or unpaired) or a Mann-Whitney-*U*-test was applied. An *F*-test decided which test method had to be used. *P* < 0.05 was considered to be statistically significant.

## Results

### Anion dependence of the propionate-induced increase in*** I***_***sc***_

**Fig. 1:**
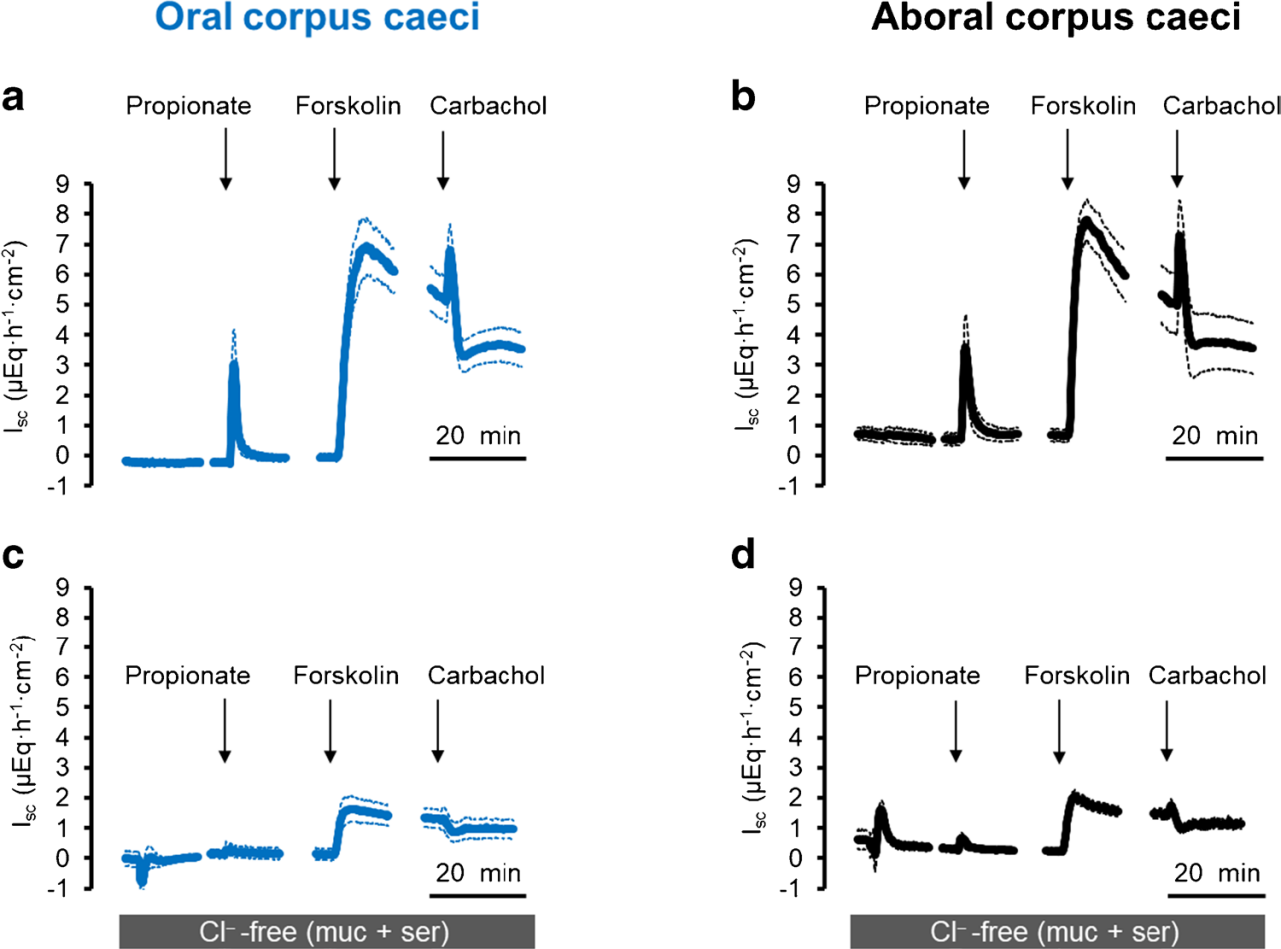
Response to Na propionate (2·10^*−*3^ mol·l^*−*1^ at the mucosal side) in the presence (**a**, **b**) and absence (**c**, **d**) of Cl^*−*^ in oral (**a**, **c**) and aboral (**b**, **d**) corpus caeci. Administration of propionate was followed by forskolin (5·10^*−*6^ mol·l^*−*1^ at the mucosal and the serosal side) and carbachol (5·10^*−*5^ mol·l^*−*1^ at the serosal side). Data are means (thick lines) ± SEM (thin lines). For *n* of the individual experimental series and statistical comparisons, see Tables 2–3. Line interruptions are caused by the omission of time intervals in order to synchronize the tracings of individual records to the administration of drugs

Propionate is known to evoke anion secretion across different segments of the large intestine from different species [32, 33], which in general is thought to be carried by Cl^−^. Indeed, when Cl^−^ was replaced on both sides of the chamber by the impermeant anion, gluconate, the increase in *I*_sc_ (*I*_Prop_) evoked by propionate (2·10^−3^ mol·l^−1^ at the mucosal side) was nearly suppressed both in the oral (Fig. 1c) as well as in the aboral (Fig. 1d) corpus caeci when compared with the *I*_Prop_ in Cl^−^-containing buffer (Fig. 1a, b). The increase in *I*_sc_ induced by propionate was concomitant with an increase in *G*_t_ (Fig. S1), which was abolished under Cl^−^-free conditions (data not shown).

Depending on the caecal segment, also the response to the cAMP-dependent secretagogue forskolin (5·10^−6^ mol·l^−1^ at the mucosal and the serosal side) was diminished significantly by 75–80% (Fig. 1, Tables 2 and 3), whereas the effect of the cholinergic agonist carbachol (5·10^−5^ mol·l^−1^ at the serosal side), administered during the plateau phase of the *I*_sc_ induced by forskolin, was even more strongly reduced under Cl^−^-free conditions. This would fit - at first glance - to the assumption that electrogenic Cl^−^ secretion underlies the propionate-induced current.

**Table 2:** Agonist-induced changes in *I*_sc_ in oral corpus caeci during ion replacement experiments

Oral corpus caeci	Propionate		Forskolin		Carbachol		*n*
	*∆I*_sc_ (μEq·h^−1^·cm^−2^)						
+ Cl^−^		3.39 ± 1.14		7.36 ± 0.89		1.76 ± 0.54	7
− Cl^−^	↓	0.25 ± 0.13*	↓	1.61 ± 0.89*	↓	0.11 ± 0.08*	7
+ Cl^−^ (muc)		2.27 ± 0.71		9.63 ± 0.46		1.76 ± 0.38	6
− Cl^−^ (muc)		4.52 ± 1.78	↓	4.85 ± 1.01*		3.19 ± 0.94	6
+ Cl^−^ (ser)		8.03 ± 0.98		7.65 ± 1.04		4.14 ± 0.77	6
− Cl^−^ (ser)	↓	0.60 ± 0.14*	↓	0.96 ± 0.27*	↓	0.80 ± 0.15*	6
+ HCO_3_^−^		4.01 ± 0.94		8.80 ± 0.49		4.74 ± 0.33	8
− HCO_3_^−^	↓	0.39 ± 0.18*	↓	4.24 ± 0.49*		3.53 ± 0.78	10
+ Na^+^ (ser)		6.38 ± 1.29		8.76 ± 0.71		3.93 ± 0.20	6
− Na^+^ (ser)	↓	0.95 ± 0.17*	↓	5.25 ± 0.24*	↓	0.72 ± 0.10*	6
+ Na^+^ (muc)		6.70 ± 0.80		8.53 ± 0.33		3.46 ± 0.32	6
− Na^+^ (muc)	↓	2.33 ± 0.97*		8.20 ± 0.40		2.60 ± 0.34	6

Increase in *I*_sc_ (∆*I*_sc_) induced by propionate (2·10^−3^ mol·l^−1^ at the mucosal side), forskolin (5·10^−6^ mol·l^−1^ at the mucosal and the serosal side) and carbachol (5·10^−5^ mol·l^−1^ at the serosal side) in the oral corpus caeci. Responses were tested in the presence and absence of Cl^−^, HCO_3_^−^, or Na^+^ on the serosal (ser) and/or the mucosal (muc) side. Values are given as difference of the maximal change in *I*_sc_ induced by the agonist (peak) and the baseline just prior to administration of the drug and are means ± SEM

^*^P < 0.05 versus response in the presence of the respective ion

**Table 3:** Agonist-induced changes in *I*_sc_ in aboral corpus caeci during ion replacement experiments

Aboral corpus caeci	Propionate		Forskolin		Carbachol		*n*
	*∆I*_sc_ (μEq·h^−1^·cm^−2^)						
+ Cl^−^		3.19 ± 0.96		7.48 ± 0.64		2.24 ± 0.48	7
− Cl^−^	↓	0.50 ± 0.14*	↓	1.91 ± 0.20*	↓	0.35 ± 0.06*	6
+ Cl^−^ (muc)		4.65 ± 1.39		7.84 ± 0.76		3.55 ± 0.92	6
− Cl^−^ (muc)		4.44 ± 1.66	↓	3.70 ± 0.90*		1.49 ± 0.25	6
+ Cl^−^ (ser)		5.19 ± 1.16		6.50 ± 1.02		4.13 ± 0.71	6
− Cl^−^ (ser)	↓	1.08 ± 0.23*	↓	0.95 ± 0.36*	↓	0.82 ± 0.19*	6
+ HCO_3_^−^		2.99 ± 0.85		8.77 ± 0.75		4.75 ± 0.64	9
− HCO_3_^−^	↓	0.36 ± 0.10*	↓	4.56 ± 0.57*		4.45 ± 0.69	10
+ Na^+^ (ser)		5.55 ± 0.78		5.92 ± 0.77		4.13 ± 0.43	6
− Na^+^ (ser)	↓	1.01 ± 0.24*		4.88 ± 0.51	↓	1.18 ± 0.29*	6
+ Na^+^ (muc)		4.73 ± 1.23		6.48 ± 2.37		3.90 ± 0.74	6
− Na^+^ (muc)		2.82 ± 0.96		6.00 ± 1.03		2.64 ± 0.38	6

Increase in *I*_sc_ (∆*I*_sc_) induced by propionate (2·10^−3^ mol·l^−1^ at the mucosal side), forskolin (5·10^−6^ mol·l^−1^ at the mucosal and the serosal side) and carbachol (5·10^−5^ mol·l^−1^ at the serosal side) in the aboral corpus caeci. Responses were tested in the presence and absence of Cl^−^, HCO_3_^−^, or Na^+^ on the serosal (ser) and/or the mucosal (muc) side. Values are given as difference of the maximal change in *I*_sc_ induced by the agonist (peak) and the baseline just prior to administration of the drug and are means ± SEM

^*^P < 0.05 versus response in the presence of the respective ion

**Fig. 2:**
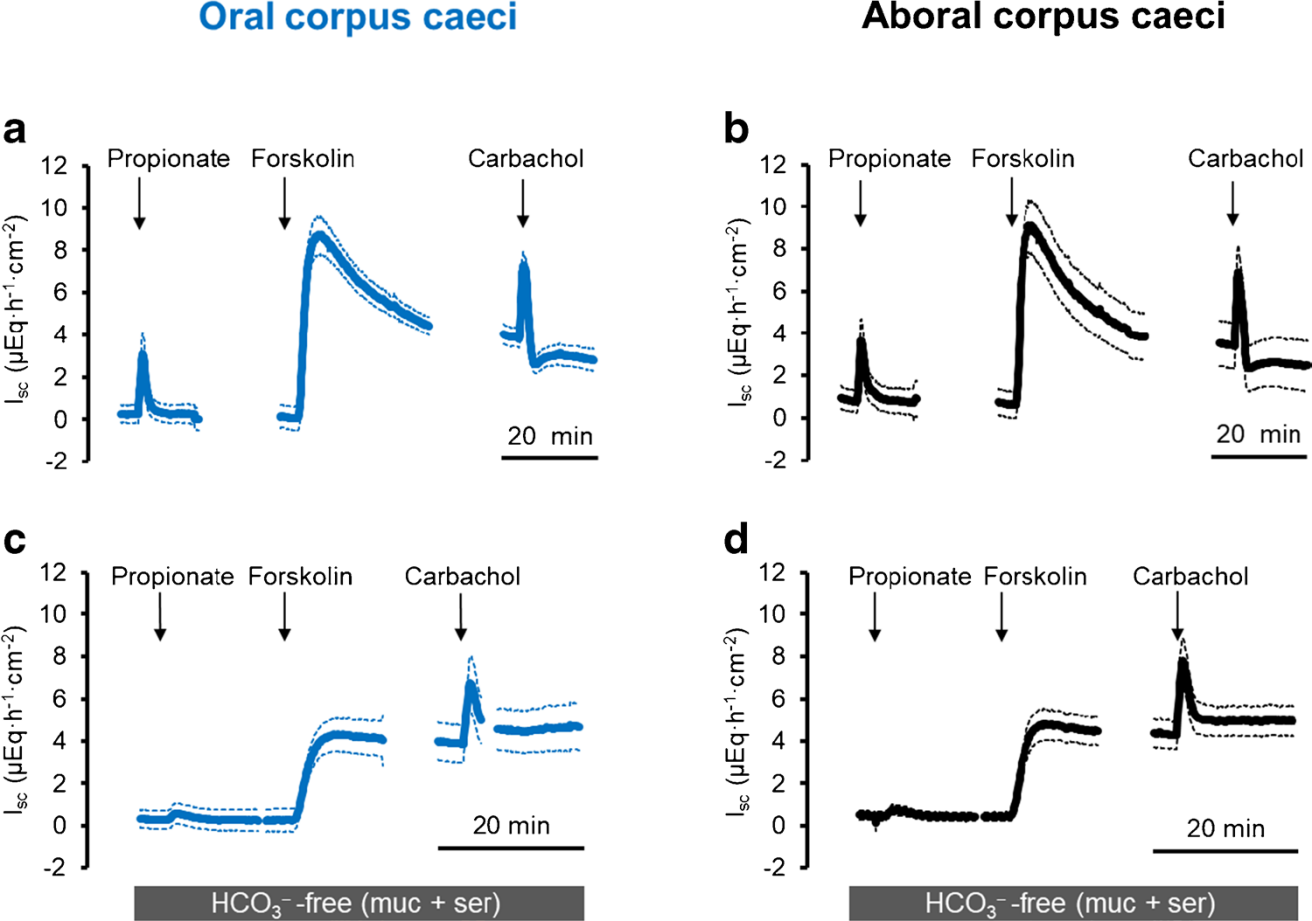
Response to Na propionate (2·10^*−*3^ mol·l^*−*1^ at the mucosal side) in the presence (**a**, **b**) or absence (**c**, **d**) of HCO_3_^*−*^ in oral (**a**, **c**) and aboral (**b**, **d**) corpus caeci. Administration of propionate was followed by forskolin (5·10^*−*6^ mol·l^*−*1^ at the mucosal and the serosal side) and carbachol (5·10^*−*5^ mol·l^*−*1^ at the serosal side). Data are means (thick lines) ± SEM (thin lines). For *n* of the individual experimental series and statistical comparisons, see Tables 2 and 3. Line interruptions are caused by the omission of time intervals in order to synchronize the tracings of individual records to the administration of drugs

The prerequisite for Cl^−^ secretion across apical anion channels is the intracellular accumulation of this anion above its electrochemical equilibrium, a process which is generally mediated by secondary active basolateral Cl^−^ uptake via the Na^+^-K^+^-2Cl^–^-cotransporter type 1 (NKCC1; [12]). Surprisingly, pretreatment with bumetanide (10^−4^ mol·l^−1^ at the serosal side; for the effect of this inhibitor and other inhibitors on baseline *I*_sc_, see Table S1), a potent blocker of NKCCs, only partially reduced *I*_Prop_ (without reaching statistical significance), whereas the response to carbachol was strongly reduced. The inhibition reached statistical significance only in the aboral corpus caeci (Table 5) but not in the oral part due to the large variability of the control group (Table 4). This suggests that the electrogenic transport of an ion other than Cl^−^ essentially contributes to the current evoked by the short-chain fatty acid. Therefore, the experiments were repeated in HCO_3_^−^-free buffer. Under these conditions, the *I*_sc_ induced by propionate (2·10^−3^ mol·l^−1^ at the mucosal side) was inhibited by about 90% in both caecal segments (*p* < 0.05). Forskolin-induced *I*_sc_ was reduced under these conditions by about 50% (*p* < 0.05), whereas the current induced by carbachol was nearly unaffected (Fig. 2, Tables 2 and 3). Blockade of carbonic anhydrases with methazolamide (10^−4^ mol·l^−1^ at the mucosal and the serosal side) did not significantly affect the *I*_sc_ induced by any of the three tested agonists nor did it enhance the inhibition of the propionate or forskolin response under HCO_3_^−^-free conditions (Tables 4 and 5), suggesting that HCO_3_^−^ produced intracellularly from the metabolism of CO_2_ does not play a role in propionate-induced *I*_sc_.

**Table 4:** Effect of drugs affecting membrane transporters on agonist-induced changes in *I*_sc_ in oral corpus caeci

Oral corpus caeci	Propionate		Forskolin		Carbachol	*n*
	*∆I*_sc_ (μEq·h^−1^ ·cm^−2^)					
With HCO_3_^−^						
− Bumetanide		1.42 ± 0.79		7.79 ± 1.69	1.12 ± 0.59	6
+ Bumetanide		0.96 ± 0.22		4.10 ± 0.21^p<0.06^	0.13 ± 0.07	6
− Methazolamide		2.40 ± 1.32		9.64 ± 0.61	4.90 ± 0.52	6
+ Methazolamide		3.17 ± 0.91		9.01 ± 1.18	4.99 ± 0.70	8
− SITS (ser)		2.93 ± 0.94		6.70 ± 0.46	1.96 ± 0.65	6
+ SITS (ser)		1.91 ± 0.39		7.35 ± 0.60	1.98 ± 0.45	6
− SITS (muc)		3.18 ± 1.26		7.44 ± 0.68	2.52 ± 0.54	7
+ SITS (muc)		6.19 ± 1.06		8.49 ± 0.73	2.99 ± 0.55	8
− DIDS (ser)		6.38 ± 1.41		9.37 ± 0.62	3.67 ± 0.98	6
+ DIDS (ser)		7.80 ± 2.08		9.55 ± 1.11	3.27 ± 0.57	6
− DIDS (muc)		3.02 ± 1.15		7.05 ± 0.56	2.64 ± 0.22	6
+ DIDS (muc)		2.35 ± 0.94		6.32 ± 0.51	2.50 ± 0.32	6
− DNDS (ser)		3.49 ± 1.59		11.13 ± 1.52	3.48 ± 0.71	7
+ DNDS (ser)	↑	8.61 ± 1.38*		10.21 ± 2.24	5.34 ± 1.11	6
− DNDS (muc)		3.84 ± 1.49		10.05 ± 0.72	3.97 ± 0.30	7
+ DNDS (muc)		3.80 ± 1.58	↓	7.69 ± 1.43*	2.46 ± 0.83	6
HCO_3_^−^ free						
− Methazolamide		0.28 ± 0.00		4.23 ± 0.16	2.13 ± 0.56	6
+ Methazolamide		0.16 ± 0.06		3.72 ± 0.86	3.05 ± 1.05	7
− 1-EBIO		0.22 ± 0.06		4.31 ± 0.69	1.81 ± 0.46	6
+ 1-EBIO		0.26 ± 0.07	↑	9.05 ± 0.66*	1.97 ± 0.68	6

Increase in *I*_sc_ (∆*I*_sc_) induced by propionate (2·10^−3^ mol·l^−1^ at the mucosal side), forskolin (5·10^−6^ mol·l^−1^ at the mucosal and the serosal side) and carbachol (5·10^−5^ mol·l^−1^ at the serosal side) in the oral corpus caeci. Responses were tested in the presence and absence of methazolamide (10^−4^ mol·l^−1^ at the mucosal and the serosal side), bumetanide (10^−4^ mol·l^−1^ at the serosal side), SITS (10^−3^ mol·l^−1^ at the serosal or the mucosal side), DIDS (10^−3^ mol·l^−1^ at the serosal or the mucosal side), or DNDS (5·10^−3^ mol·l^−1^ at the serosal or the mucosal side). In two series of experiments, the response to the agonists was tested in the absence of HCO_3_^–^ combined with the absence or presence of 1-EBIO (2·10^−3^ mol·l^−1^ at the serosal side) or methazolamide (10^−4^ mol·l^−1^ at the mucosal and the serosal side). If the drugs to be tested were not dissolved in aqueous stock solutions, the control tissues were treated with the same volume of the respective solvent (DMSO, ethanol). Values are given as difference of the maximal change in *I*_sc_ induced by the agonist (peak) and the baseline just prior to administration of the drug and are means ± SEM

^*^P < 0.05 versus response in the absence of the respective drug

**Table 5:** Effect of drugs affecting membrane transporters on agonist-induced changes in *I*_sc_ in aboral corpus caeci

Aboral corpus caeci	Propionate		Forskolin		Carbachol		*n*
	*∆I*_sc_ (μEq·h^−1^ ·cm^−2^)						
With HCO_3_^−^							
− Bumetanide		3.37 ± 0.89		6.88 ± 1.65		2.85 ± 0.37	6
+ Bumetanide		1.64 ± 0.42		3.78 ± 0.32	↓	0.30 ± 0.14*	6
− Methazolamide		4.87 ± 1.66		8.74 ± 0.80		4.74 ± 0.47	6
+ Methazolamide		2.18 ± 0.55		6.49 ± 0.98		4.13 ± 0.35	6
− SITS (ser)		3.29 ± 1.02		5.57 ± 0.79		2.12 ± 0.50	6
+ SITS (ser)		4.03 ± 1.10		4.99 ± 0.75		3.07 ± 0.28	6
− SITS (muc)		7.15 ± 1.06		7.35 ± 0.70		2.99 ± 0.50	8
+ SITS (muc)	↓	3.25 ± 0.62*		6.85 ± 1.07		2.46 ± 0.50	7
− DIDS (ser)		4.99 ± 1.42		8.41 ± 0.64		2.40 ± 0.53	6
+ DIDS (ser)		5.99 ± 1.37		8.17 ± 0.51		3.00 ± 0.63	6
− DIDS (muc)		4.56 ± 0.84		5.35 ± 0.46		3.06 ± 0.24	6
+ DIDS (muc)	↓	1.89 ± 0.50*		5.05 ± 0.51		3.16 ± 0.27	6
− DNDS (ser)		6.11 ± 2.86		9.66 ± 1.34		4.28 ± 1.71	6
+ DNDS (ser)		4.81 ± 1.01	↓	6.45 ± 1.05*		4.96 ± 0.45	6
− DNDS (muc)		6.56 ± 0.85		8.80 ± 0.38		4.29 ± 0.41	7
+ DNDS (muc)	↓	2.91 ± 0.75*	↓	5.99 ± 0.78*	↓	2.98 ± 0.62*	7
HCO_3_^−^ free							
− Methazolamide		0.27 ± 0.05		4.04 ± 0.45		3.98 ± 0.70	7
+ Methazolamide		0.29 ± 0.06		4.43 ± 0.49		4.44 ± 0.86	7
− 1-EBIO		0.47 ± 0.30		4.26 ± 0.65		2.34 ± 0.63	6
+ 1-EBIO		0.64 ± 0.38	↑	7.94 ± 0.48*		1.95 ± 0.83	6

Increase in *I*_sc_ (∆*I*_sc_) induced by propionate (2·10^−3^ mol·l^−1^ at the mucosal side), forskolin (5·10^−6^ mol·l^−1^ at the mucosal and the serosal side), and carbachol (5·10^−5^ mol·l^−1^ at the serosal side) in the oral corpus caeci. Responses were tested in the presence and absence of methazolamide (10^−4^ mol·l^−1^ at the mucosal and the serosal side), bumetanide (10^−4^ mol·l^−1^ at the serosal side), SITS (10^−3^ mol·l^−1^ at the serosal or the mucosal side), DIDS (10^−3^ mol·l^−1^ at the serosal or the mucosal side), or DNDS (5·10^−3^ mol·l^−1^ at the serosal or the mucosal side). In two series of experiments, the response to the agonists was tested in the absence of HCO_3_^–^ combined with the absence or presence of 1-EBIO (2·10^−3^ mol·l^−1^ at the serosal side). If the drugs to be tested were not dissolved in aqueous stock solutions, the control tissues were treated with the same volume of the respective solvent (DMSO, ethanol). Values are given as difference of the maximal change in *I*_sc_ induced by the agonist (peak) and the baseline just prior to administration of the drug and are means ± SEM

^*^P < 0.05 versus response in the absence of the respective drug

### Effect of stilbene derivatives

**Fig. 3:**
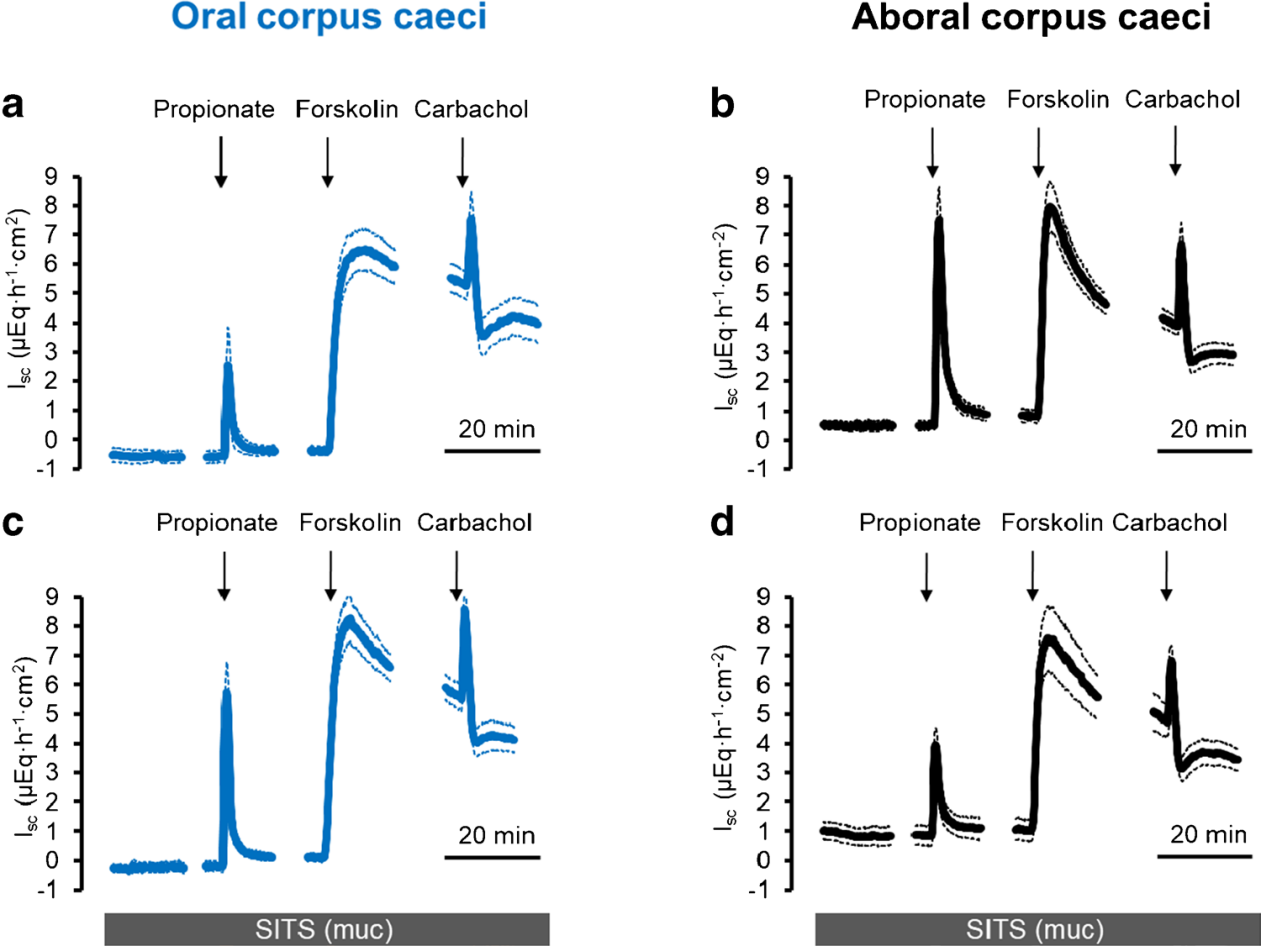
Response to Na propionate (2·10^*−*3^ mol·l^*−*1^ at the mucosal side) in the presence of HCO_3_^*−*^ and in the absence (**a**, **b**) or presence (**c**, **d**) of SITS (10^*−*3^ mol·l^*−*1^ at the mucosal side) in oral (**a**, **c**) and aboral (**b**, **d**) corpus caeci. Administration of propionate was followed by forskolin (5·10^*−*6^ mol·l^*−*1^ at the mucosal and the serosal side) and carbachol (5·10^*−*5^ mol·l^*−*1^ at the serosal side). Data are means (thick lines) ± SEM (thin lines). For *n* of the individual experimental series and statistical comparisons, see Tables 4 and 5. Line interruptions are caused by the omission of time intervals in order to synchronize the tracings of individual records to the administration of drugs

The electrogenic response evoked by propionate (*I*_Prop_) is dependent on Cl^−^ and HCO_3_^−^, which suggests the involvement of transporters for both anions, such as Cl^−^/HCO_3_^−^ exchangers. Possible action sites could be the basolateral membrane, where these exchangers might mediate Cl^−^ uptake, or the apical membrane, where such an exchanger could work in parallel with a Cl^−^ channel to mediate HCO_3_^−^ efflux [29]. Consequently, different stilbenes, which are prototypical (albeit nonselective) blockers of Cl^−^/HCO_3_^−^ exchangers, were tested. In the oral corpus caeci, none of the three tested stilbenes, i.e., SITS (10^−3^ mol·l^−1^), DIDS (10^−3^ mol·l^−1^), or DNDS (5·10^−3^ mol·l^−1^), applied either on the mucosal or the serosal side, inhibited significantly *I*_Prop_. The only effect observed was a paradox enhancement of the current induced by the short-chain fatty acid after pretreatment with serosal DNDS (Table 4). In contrast, in the aboral corpus caeci, all three stilbenes, when administered at the mucosal side, inhibited *I*_Prop_ by about 55% (*p* < 0.05; Fig. 3, Table 5) indicating a segmental difference in the transport mechanisms in the apical membrane in both caecal segments.

### Na^+^ dependence

**Fig. 4:**
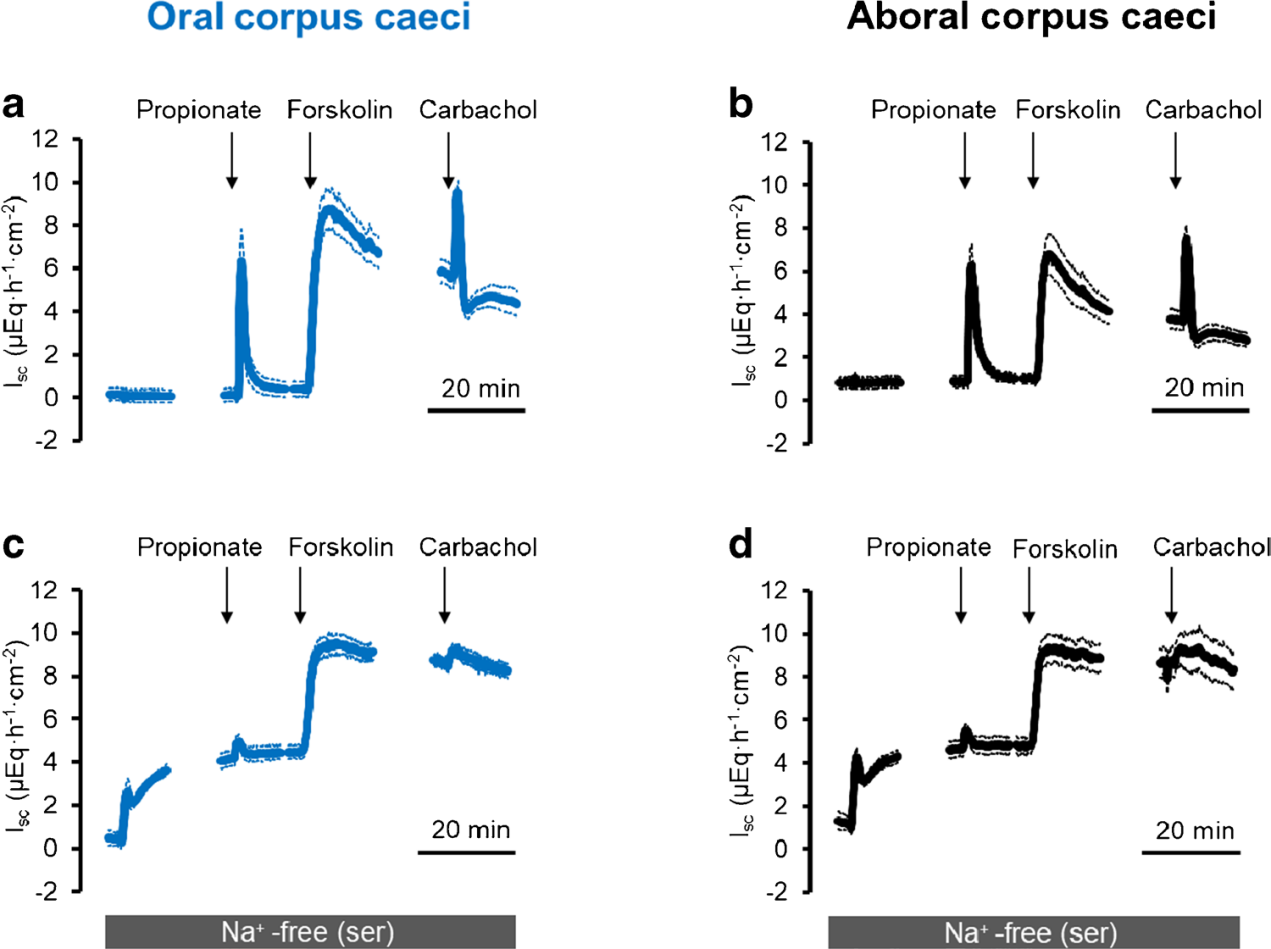
Response to Na propionate (2·10^*−*3^ mol·l^*−*1^ at the mucosal side) in the presence (**a**, **b**) and the absence (**c**, **d**) of serosal Na^+^ in oral (**a**, **c**) and aboral (**b**, **d**) corpus caeci. Administration of propionate was followed by forskolin (5·10^*−*6^ mol·l^*−*1^ at the mucosal and the serosal side) and carbachol (5·10^*−*5^ mol·l^*−*1^ at the serosal side). Data are means (thick lines) ± SEM (thin lines). For *n* of the individual experimental series and statistical comparisons, see Tables 2 and 3. Line interruptions are caused by the omission of time intervals in order to synchronize the tracings of individual records to the administration of drugs

HCO_3_^−^ uptake into cells can also be mediated by neutral (NBCn) or electrogenic (NBCe) Na^+^-HCO_3_^−^-cotransporters [26]. Consequently, the dependence of the propionate-induced current on the presence of serosal Na^+^ was tested. Indeed, in the absence of serosal Na^+^, the propionate-induced *I*_sc_ was inhibited by 80–85% in both caecal segments (*p* < 0.05, Fig. 4, Tables 2 and 3). As was the case with bumetanide, forskolin-induced *I*_sc_ was inhibited only by 40% (*p* < 0.05,Table 2) in the oral and only numerically decreased by about 20% in the aboral corpus caeci (Table 3) suggesting that further transporters besides the Na^+^-dependent NKCC1 must be involved in basolateral anion uptake during cAMP-activated secretion. In sharp contrast, carbachol-induced *I*_sc_ was inhibited by more than 70–80% in both caecal segments (*p* < 0.05, Tables 2 and 3). Interestingly, mucosal Na^+^-free conditions also inhibited *I*_Prop_ in the oral segment by 65% (*p* < 0.05, Table 2) and reduced this current numerically by 40% in the aboral segment (not significant, Table 3).

The dominant anion channel in the intestinal epithelium, the CFTR, is permeable to Cl^−^ but also to a lesser degree to HCO_3_^−^ [24]. An increased Cl^−^ secretion has been observed after stimulation of the epithelium with forskolin or the acetylcholine derivate carbachol (Figs. 1) and 2), whereas challenging of the tissue with propionate (and the release of epithelial acetylcholine, see also [32, 33]) led to an enhanced HCO_3_^−^ secretion. Thus, the question arises of how the CFTR can switch from a Cl^−^ to a HCO_3_^−^ secretion while challenging with different secretagogues.

In the human airway cell line, Calu-3, this switch has been shown to be caused by the membrane potential, as hyperpolarization of the basolateral membrane favors the uptake of HCO_3_^−^ via NBCe and thus causes a switch in the transepithelial secretion from Cl^−^ to HCO_3_^−^ [11]. Consequently, we tested whether the activation of basolateral Ca^2+^-dependent K^+^ channels by 1-EBIO might overcome the strong HCO_3_^−^-dependence of the propionate-induced current. 1-EBIO (2·10^−3^ mol·l^−1^) caused a prolonged increase in *I*_sc_ of 0.80 ± 0.17 μEq·h^−1^·cm^−2^ (*n* = 6) in the oral and of 1.60 ± 0.99 μEq·h^−1^·cm^−2^ (*n* = 6) in the aboral corpus caeci. However, the effect of propionate under HCO_3_^−^-free conditions was not enhanced in either segment. Effectivity of the long-term activation of Ca^2+^-dependent K^+^ channels was demonstrated by the significant enhancement of forskolin-induced *I*_sc_, which was roughly doubled in both caecal segments (Tables 4 and 5).

### Propionate-stimulated currents across the basolateral membrane

**Fig. 5:**
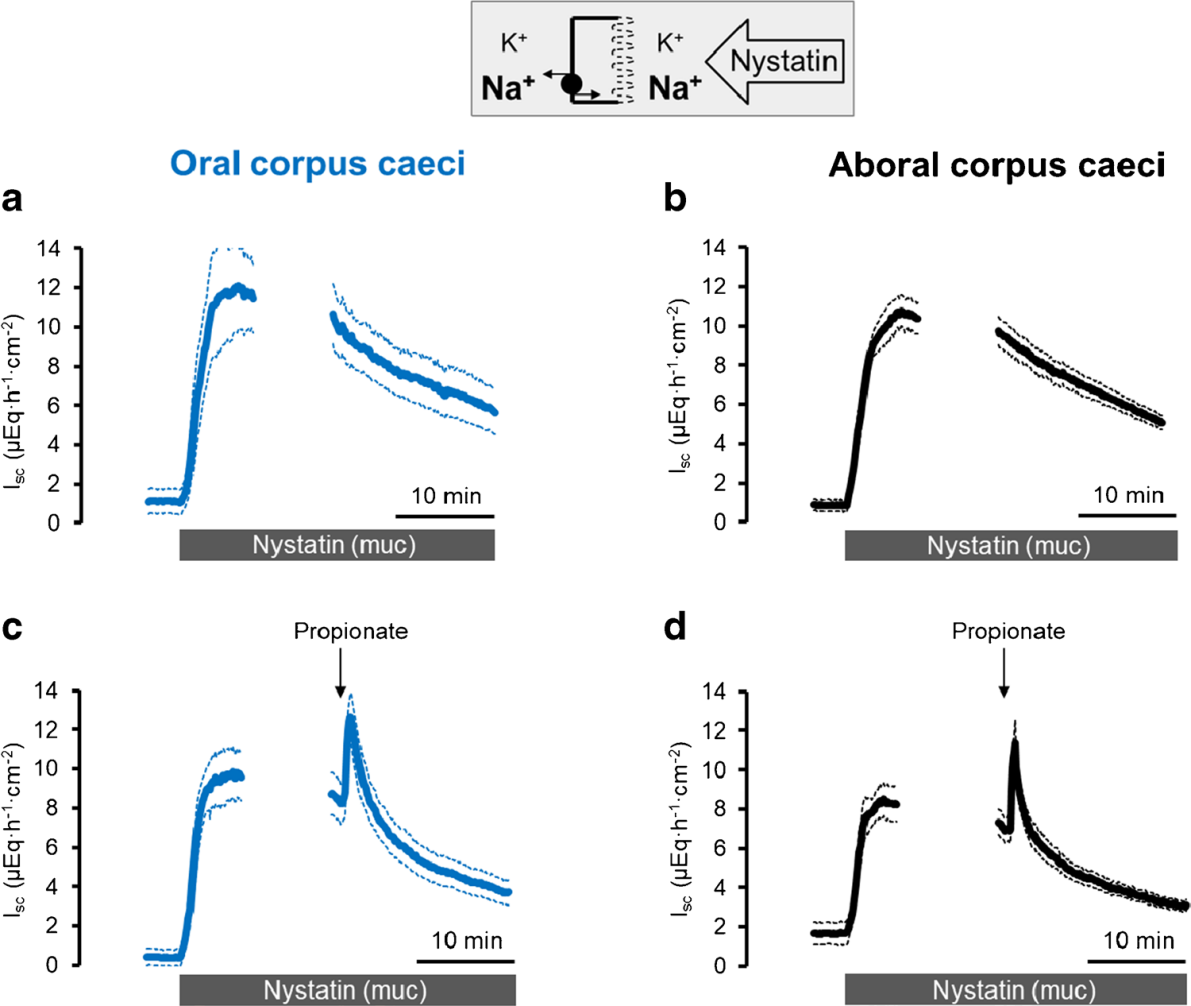
Response to Na propionate (2·10^*−*3^ mol·l^*−*1^ at the mucosal side) after permeabilization of the apical membrane with nystatin (100 μg·ml^*−*1^ at the mucosal side) on current carried by Na^+^-K^+^-pump across the basolateral membrane (107 NaCl/4.5 KCl at the mucosal and the serosal side) in comparison to time-dependent controls (**a**, **b**) in oral (**a**, **c**) and aboral (**b**, **d**) corpus caeci. Data are means (thick lines) ± SEM (thin lines). For *n* of the individual experimental series and statistical comparisons, see Tables 6 and 7. Line interruptions are caused by the omission of time intervals in order to synchronize the tracings of individual records to the administration of propionate

**Fig. 6:**
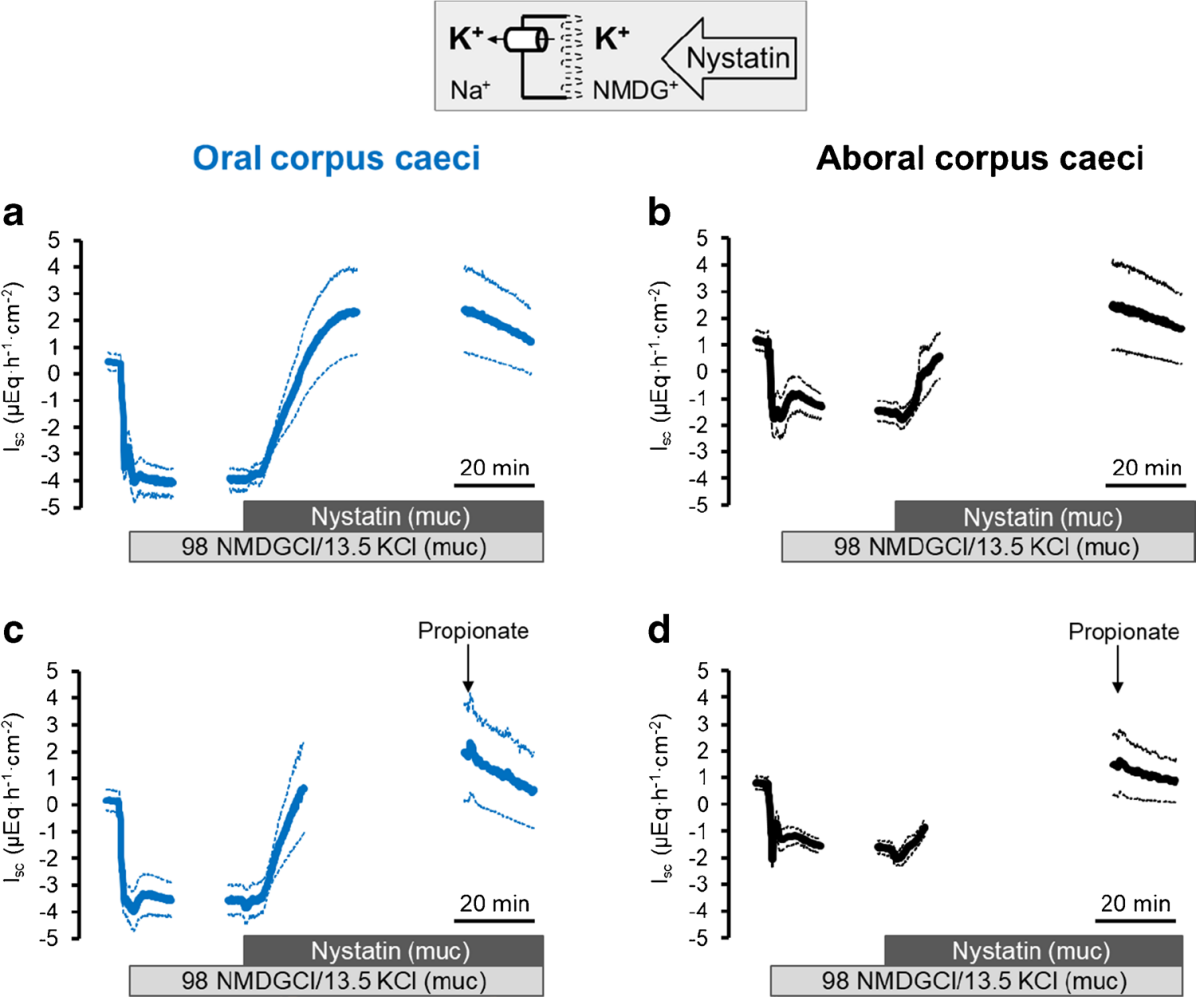
Response to Na propionate (2·10^*−*3^ mol·l^*−*1^ at the mucosal side; **c**, **d**) after permeabilization of the apical membrane with nystatin (100 μg·ml^*−*1^ at the mucosal side) on K^+^ channel current across the basolateral membrane (107 NaCl/4.5 KCl at the serosal side; 98 NMDGCl/13.5 KCl at the mucosal side) in comparison to time-dependent controls (**a**, **b**) in oral (**a**, **c**) and aboral (**b**, **d**) corpus caeci. Data are means (thick lines) ± SEM (thin lines). For *n* of the individual experimental series and statistical comparisons, see Tables 6 and 7. Line interruptions are caused by the omission of time intervals in order to synchronize the tracings of individual records to the administration of propionate

The *I*_sc_ induced by propionate in the caecum [6] is - similar as in other segments of the large intestine ([32, 33] - thought to be mediated by the release of epithelial acetylcholine as evidenced, e.g., by its sensitivity to the muscarinic receptor blocker atropine and simultaneous resistance against the neurotoxin tetrodotoxin. The dominant action site of acetylcholine is the basolateral membrane, as stimulation of cholinergic receptors on the epithelium stimulates Ca^2+^-dependent K^+^ channels [27] and increases the current caused by the activity of the Na^+^-K^+^-pump [7]. In order to investigate potential changes in electrogenic ion transport across the basolateral membrane, propionate-induced currents across this membrane were studied after permeabilization of the apical membrane with nystatin. Different ionic conditions were applied to selectively measure pump currents and currents across basolateral K^+^ channels. With symmetrical Na^+^ concentrations and in the absence of a chemical K^+^ gradient, the permeabilization of the apical membrane leads to a massive increase in Na^+^-K^+^-pump activity (and thereby an increase in *I*_sc_) due to the influx of Na^+^ into the cells via the nystatin pores (Fig. 5, Table 6). This pump current was significantly increased when propionate (2·10^−3^ mol·l^−1^ at the mucosal side) was administered during the decaying phase of the nystatin-induced *I*_sc_ (*p* < 0.05; Fig. 5, Table 7). In contrast, when nystatin was applied under mucosal Na^+^-free conditions (to avoid currents by the Na^+^-K^+^-pump) with a 3:1 K^+^ concentration gradient (13.5 mmol·l^−1^ at the mucosal and 4.5 mmol·l^−1^ at the serosal side as depicted in the inset of Fig. 6) to drive K^+^ currents across the basolateral membrane, no significant change in *I*_sc_ across the basolateral membrane was measured after administration of propionate (Fig. 6, Table 7).

**Table 6:** Currents across the basolateral membrane

Caecum	Nystatin-induced *I*_sc_ (∆*I*_sc_; μEq·h^−1^·cm^−2^)		*n*
	Oral	Aboral	
*I*_Pump_	11.35 ± 3.15	9.18 ± 2.55	13/13
*I*_K+ channels_	2.14 ± 0.59	1.38 ± 0.38	13/13
*I*_Remain_	1.89 ± 0.55	1.64 ± 0.47	12/12

Currents across the basolateral membrane induced by permeabilization of the apical membrane with nystatin (100 μg·ml^−1^ at the mucosal side). Ionic conditions to measure the individual currents were the following:

pump current: mucosal 107 mM NaCl, 4.5 KCl; serosal 107 mM NaCl, 4.5 KCl. K^+^ channel current: mucosal 98 mM NMDGCl, 13.5 KCl; serosal 107 mM NaCl, 4.5 KCl. Remaining currents: mucosal 107 mM NMDGCl, 4.5 KCl; serosal 107 mM NaCl, 4.5 KCl. Values are given as difference of the maximal change in *I*_sc_ induced by nystatin (peak) and the baseline just prior administration of the ionophore and are means ± SEM

**Table 7:** Propionate-induced currents across the basolateral membrane

Caecum	Propionate-induced *I*_sc_ (∆*I*_sc_; μEq·h^−1^·cm^−2^)		*n*
	Oral	Aboral	
*I*_Pump_	5.57 ± 0.59*	5.69 ± 0.84*	7/7
*I*_K+ channels_	0.17 ± 0.14	0.15 ± 0.06	7/6
*I*_Remain_	1.14 ± 0.39*	0.81 ± 0.25	6/6

Effect of propionate (2·10^−3^ mol·l^−1^ at the mucosal side) on currents across the basolateral membrane. Ionic conditions were the following:

pump current: mucosal 107 mM NaCl, 4.5 KCl; serosal 107 mM NaCl, 4.5 KCl

K^+^ channel current: mucosal 98 mM NMDGCl, 13.5 KCl; serosal 107 mM NaCl, 4.5 KCl

Remaining currents: mucosal 107 mM NMDGCl, 4.5 KCl; serosal 107 mM NaCl, 4.5 KCl

Values are given as difference to the extrapolated current calculated from the current decay just prior to administration of propionate (∆*I*_sc_) and are means ± SEM

^*^P < 0.05 versus *I*_sc_ course in time-dependent controls (*n* = 6–8), which were analyzed according to the same schedule used to identify the peak current induced by propionate (see Methods)

Surprisingly, when propionate was administered under conditions when no pump current should be possible (NMDG^+^ instead of Na^+^ at the mucosal side) and without a chemical K^+^ gradient to drive K^+^ currents, propionate induced a visible increase in *I*_sc_ that tested for significance in the oral corpus caeci (Table 7; see Discussion).

### Expression of Na^+^-HCO_3_^−^-cotransporters

**Fig. 7:**
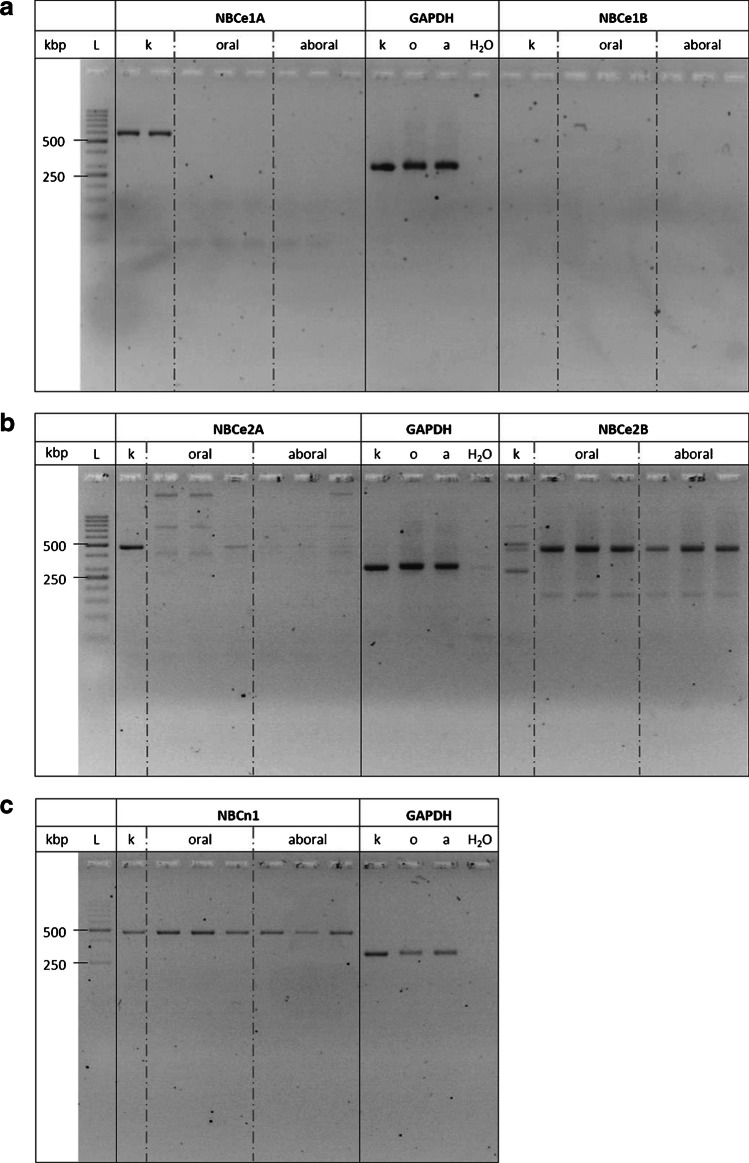
cDNA prepared by RT-PCR using primers specific for NBCe1A or NBCe1B (**a**), NBCe2A or NBCe2B (**b**), and NBCn1 (**c**). Primer-free PCR (“H_2_O”) did not reveal any products. Homogenates from rat kidney served as reference tissue to check the efficiency of the selected primers. GAPDH was used as “housekeeping gene” to check the quality of the PCR reaction. The DNA ladders (“L”) contained cDNA from 50 to 1000 bp in 50-bp steps (from 50 to 300 bp) or 100-bp steps (from 300 to 1000 bp), respectively. Expected product sizes were NBCe1A: 597 bp, NBCe1B: 724 bp, NBCe2A: 477 bp, NBCe2B 503 bp, NBCn1: 477 bp, GAPDH: 303 bp. Representative picture from three independent experiments with similar results

Because of the strong Na^+^ dependence of basolateral anion uptake into the caecal epithelium, which seems not only to be mediated by the NKCC1 (Fig. 4, Tables 2 and 4), the expression of neutral (NBCn) and electrogenic (NBCe) Na^+^-HCO_3_^–^-cotransporters in the oral and aboral part of the caecum was measured with RT-PCR. Neither in the oral nor in the aboral segment of the caecum the cDNA of the electrogenic cotransporters NBCe1A or NBCe2A could be found, whereas both transporters are expressed in the kidney which was used as reference tissue (Fig. 7). No expression in all of the tested tissues could be observed for the NBCe1B, although the expression of the reference gene GAPDH was found in the kidney (Fig. 7). Interestingly, as in the kidney, the neutral cotransporter NBCn1 was robustly expressed in the oral and in the aboral segment at the expected size of 477 bp (Fig. 7). For the electrogenic transporter NBCe2B, cDNA expression was observed in both parts of the caecum as well as in the kidney (Fig. 7). The presence of this electrogenic transporter could explain the remaining current at the basolateral membrane, which is independent of the pump and K^+^ currents (Table 7; see Discussion).

## Discussion

The present experiments demonstrate that propionate-induced *I*_sc_, which has been shown to be mediated by the release of non-neuronal acetylcholine from the intestinal epithelium [32, 33], differs from “classical” intestinal Cl^−^ secretion. This is thought to be mediated by intracellular accumulation of Cl^−^ above its electrochemical equilibrium followed by apical efflux across an anion channel [13]. This process centrally involves basolateral Cl^−^ uptake via NKCC1 (for a recent review see [12]). In the case of rat caecum, the increase in short-circuit current induced by propionate is sensitive to atropine but resistant to tetrodotoxin [6], i.e., mediated by the release of acetylcholine from epithelial cells. Indeed, the expression of the key enzyme for acetylcholine synthesis, the choline acetyltransferase (ChAT), is found in the epithelium with a higher expression level in the aboral compared to the oral corpus caeci [6]. However, the propionate-induced *I*_sc_ is only partially sensitive to bumetanide, a prototypical blocker of NKCCs ([6], and Tables 4 and 5 for the present study), and is mutually dependent on the presence of Cl^−^ as well as HCO_3_^−^ (Figs. 1 and 2, Tables 2 and 3), which does not fit to the classical model for Cl^−^ secretion.

**Fig. 8:**
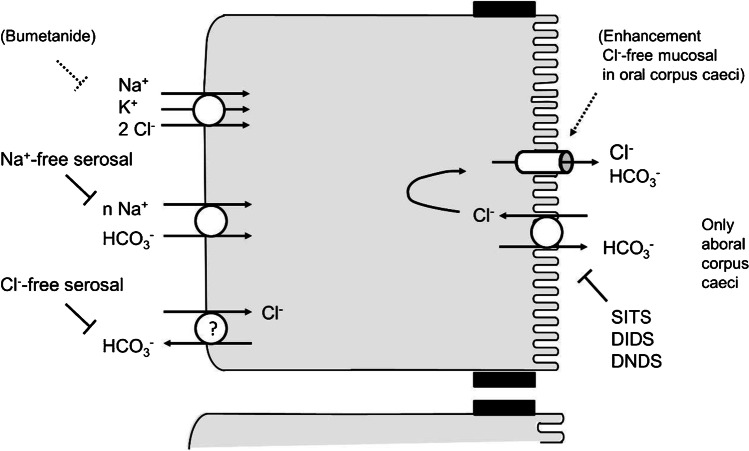
Model with the presumed transporters involved in anion secretion evoked by propionate in rat caecum. Significant effects of inhibitors and ion substitution experiments are printed with intact lines, tendencies not reaching statistical significances in the present experiments (but in a previously published study in the case of bumetanide) with dotted lines

A plausible explanation for the ionic mechanisms underlying *I*_Prop_ is that the bumetanide-resistant part of the current involves the transport of HCO_3_^−^. One functional group of transporters to be discussed are Cl^−^/HCO_3_^−^ exchangers belonging to the class of SLC (solute carrier) 4 proteins such as AE1 (SLC4A1), AE2 (SLC4A2), and AE3 (SLC4A3) [21, 26], or SLC26 proteins [1] such as DRA (SLC26A3; downregulated in adenoma). Basolateral Cl^−^/HCO_3_^−^ exchanger(s) are found, e.g., in the colonic epithelium [5, 14]; they could mediate uptake of Cl^−^ to be secreted via apical anion channels. Apical Cl^−^/HCO_3_^−^ antiporters can work in parallel with a Cl^−^ channel to mediate HCO_3_^−^ efflux as shown, e.g., for duodenum, where HCO_3_^−^ secretion is an essential protective mechanism against gastric HCl [29]. Consequently, the sensitivity of *I*_Prop_ to different stilbene derivates, which are nonselective blockers of different anion transporters including Cl^−^/HCO_3_^−^ exchangers [10], was tested. When administered on the mucosal side of the aboral caecal epithelium, all three stilbene derivatives tested, i.e., SITS (Fig. 3), DIDS, and DNDS, inhibited *I*_Prop_ significantly by about 55% (Table 5), which would be in accordance with the assumption that an apical, stilbene-sensitive transport process mediating Cl^−^/HCO_3_^−^ exchange is involved in propionate-induced anion (HCO_3_^−^) secretion. In the oral segment, these blockers did not inhibit *I*_Prop_ (Table 4) indicating a segmental difference within the caecum. Interestingly, *I*_Prop_ is larger in the aboral than in oral corpus caeci as reported previously [6]. This observation was confirmed in the present study: When all control series of Tables 2–5, which were performed in standard, i.e., HCO_3_^−^-containing buffer, were averaged, *I*_Prop_ amounted to 4.08 ± 0.38 μEq·h^−1^·cm^−2^ (*n* = 82) in the oral and 4.99 ± 0.32 μEq·h^−1^·cm^−2^ (*n* = 82, *p* < 0.05 versus response in the oral corpus caeci) in the aboral part, whereas the increase in *G*_t_ after propionate administration did not differ significantly between the oral (3.02 ± 0.28 mS cm^−2^, *n* = 82) and the aboral corpus caeci (3.99 ± 0.80 mS·cm^−2^, *n* = 82). This would be compatible with the assumption that an additional secretory pathway (i.e., an anion exchanger in the apical membrane side working in parallel with an apical anion conductance) is activated in the aboral part of the caecum. This would also explain why in the oral corpus caeci *I*_Prop_ tended to be enhanced under mucosal Cl^−^-free conditions (Table 2), which should increase the driving force for Cl^−^ efflux across the apical membrane. However, *I*_Prop_ was slightly decreased in the aboral segment under mucosal Cl^−^-free conditions (Table 3), where the expected stimulation of *I*_sc_ via increased flux across apical anion channels might be counteracted by interrupting the cycling of chloride between apical Cl^−^/HCO_3_^−^ exchangers and apical anion channels (Fig. 8).

Regarding the basolateral membrane, where the transporters loading the epithelium with the respective anions for Cl^−^ or HCO_3_^−^ secretion are located, none of the tested stilbenes had any inhibitory effect on propionate-induced current (Tables 4 and 5). However, there was a strong inhibition of *I*_Prop_ in both caecal segments in the absence of serosal Cl^−^ and in the absence of serosal Na^+^ (Tables 2 and 3). The latter might be well explained by Na^+^-HCO_3_^−^-cotransporters in the basolateral membrane, which are also involved, e.g., in duodenal HCO_3_^−^ secretion [30]. Indeed, several members of this group of cotransporters, i.e., NBCe2B and NBCn1, were found to be expressed both in the oral as well as the aboral part of the caecum (Fig. 7). It is known that these transporters are often but not always inhibited by stilbenes [26], which did, however, not affect *I*_Prop_ when applied from the serosal side (Tables 4 and 5). This might either suggest that stilbene-insensitive forms of these members of the SLC4 family of proteins are involved or that the tunica muscularis and submucosa build up a diffusion barrier preventing the access of the stilbenes in sufficient concentrations to their action sites. Such a diffusion barrier might also explain that the effect of serosal Cl^−^-free solution, which should prevent HCO_3_^−^ uptake via basolateral Cl^−^/HCO_3_^−^ antiporters is not mimicked by serosal stilbenes. Indeed, basolateral Cl^−^/HCO_3_^−^ exchanger activity has been measured in a closely related segment of the large intestine, i.e., rat colon [14].

In previous experiments using basolaterally depolarized epithelia, electrogenic transport processes in the apical membrane of the caecal epithelium were characterized. It turned out that the membrane of this scarcely investigated intestinal segment possesses cAMP- and Ca^2+^-dependent Cl^−^ conductance(s), a Ca^2+^-dependent K^+^ conductance and a conductance for Na^+^, probably mediated by nonselective cation channels [22]. In the present study, electrogenic transport and its regulation by propionate were studied after permeabilization of the apical membrane. These experiments revealed that propionate activated the current carried by the 3 Na^+^-2 K^+^-pump (Fig. 5) but had no significant effect on currents across the basolateral membrane when ionic conditions were used to measure currents across K^+^ channels (Fig. 6, Table 7). A similar stimulation of a pump current by acetylcholine has recently been shown in rat colon [7]. Consequently, activation of the 3 Na^+^-2 K^+^-pump, the “motor” for most transepithelial transport processes, is a further mechanism by which propionate may evoke anion secretion. This would involve enhancement of the chemical driving force for Na^+^-dependent secondary active transporters or enhancement of the electric driving force (via hyperpolarization of the membrane) for anion efflux via anion channels. Interestingly, even in the absence of mucosal Na^+^ (to suppress currents by the 3 Na^+^-2 K^+^-pump) and in the absence of a K^+^ concentration gradient (to suppress currents across basolateral K^+^ channels), propionate still induced an increase in *I*_sc_ in apically permeabilized epithelia (*I*_Remain_ in Table 7). This would fit to the activity of an electrogenic Na^+^-n HCO_3_^−^ cotransporter such as NBCe2B (Fig. 7) in the basolateral membrane [31], although this hypothesis is difficult to test due to the lack of specific inhibitors.

From a functional point of view, a strong contribution of HCO_3_^−^ to propionate-induced anion secretion can fulfill an important physiological function. In herbivorous animals, which use the forestomach system as a fermentation chamber, the uncontrolled production of short-chain fatty acids, observed, e.g., after feeding of non-adapted animals with an easily fermentable carbohydrate such as starch, leads to ruminal acidosis. The fall in ruminal pH severely affects the microbial ecosystem in the fermentation chamber with a loss of biodiversity in the microbial community, a proliferation of microbes producing lactic acid and a reduced number of microbes metabolizing lactic acid [4, 17]. Under healthy conditions, this disturbance of the ecosystem is prevented by buffer bases such as HCO_3_^−^ and phosphate from the salivary glands or HCO_3_^−^ transported into the ruminal lumen by HCO_3_^−^/short-chain fatty acid anion exchangers in the apical membrane of the ruminal epithelium [3, 31]. Transferring this situation to the caecum, i.e., the largest fermentation chamber of non-ruminant animals [20], caecal production of short-chain fatty acids can vary in wide bounds depending on the feeding situation [2]. An increase in the concentration of propionate, which is - together with acetate and butyrate - one of the three main short-chain fatty acids produced during microbial fermentation, would be answered by the epithelium by enhanced secretion of a buffer base. In the large intestine, there is a fine balance between lactic acid-producing bacteria (such as *Bifidobacterium* in men) and lactic acid metabolizing bacteria (such as *Eubacterium hallii* or *Roseburia hominis* in men [15]). Thus, the release of epithelial acetylcholine after binding of propionate to epithelial short-chain fatty acid receptors coupled to HCO_3_^−^ secretion is a further example for the communication between gut microbiota and the host, i.e., the mammal.

In summary, the present data show that anion secretion induced by propionate across caecal epithelium involves transporters, which mediate the secretion of both anions, Cl^−^ and HCO_3_^−^. HCO_3_^−^, in addition to Cl^−^, would not only act as an osmotically active ion (to drive water flux) but also as the buffering anion. The different sensitivity to apically administered stilbenes reveals a segmental difference between the oral and the aboral part (Fig. 8). The aboral part of the caecum, which is located close to the blind end of this organ, also exhibits a higher expression of non-neuronal acetylcholine and a larger secretory response to acetylcholine derivative, carbachol [6]. This suggests that the more pronounced contact with short-chain fatty acids, e.g., propionate-induced HCO_3_^−^ secretion, led to functional adaptation and regional differentiation in this fermentation chamber.

## Supplementary Information

Below is the link to the electronic supplementary material.

Supplementary file1 (DOCX 83 KB)
