# Supplementary material for: The role of HCO3– in propionate-induced anion secretion across rat caecal epithelium
Source: Pflugers Arch. 2021 Apr 29;473(6):937–51. doi: 10.1007/s00424-021-02565-8 (PMC8164622; doi:10.1007/s00424-021-02565-8)
Supplement: Supplementary file 1 — Supplementary file1 (DOCX 83 KB) [file 424_2021_2565_MOESM1_ESM.docx]

# Supplementary data

## **Table S1:** Effect of different drugs used on baseline I_sc_.

|  | | **Oral caecum** | **Aboral caecum** | **n** |
| --- | --- | --- | --- | --- |
|  |  | *∆I_sc_ (µEq.h^-1^.cm^-2^)* | | |
| With HCO_3_^‒^ | |  |  |  |
|  | Bumetanide (ser) | -0.20 ± 0.06* | -0.72 ± 0.12* | 6/6 |
|  | Methazolamide | 0.03 ± 0.15 | 0.11 ± 0.05 | 8/6 |
|  | SITS (ser) | 0.20 ± 0.07* | 0.18 ± 0.09 | 6/6 |
|  | SITS (muc) | 0.04 ± 0.06 | -0.17 ± 0.04* | 8/7 |
|  | DIDS (ser) | 0.46 ± 0.05* | 0.50 ± 0.14* | 6/6 |
|  | DIDS (muc) | -0.64 ± 0.05* | -0.61 ± 0.04* | 6/6 |
|  | DNDS (ser) | 1.43 ± 0.44* | 1.73 ± 0.39* | 6/6 |
|  | DNDS (muc) | 0.13 ± 0.41 | -0.42 ± 0.47 | 6/7 |
| HCO_3_^‒^-free | | | | |
|  | Methazolamide | -0.02 ± 0.07 | -0.16 ± 0.07* | 7/7 |
|  | 1-EBIO (ser) | 0.49 ± 0.16* | 1.20 ± 0.51^p=0.06^ | 6/6 |

**Table S1.** Effect of different drugs on basal short circuit currents (∆I_sc_) were measured 5 min after administration of the respective drug over a period of 3 min in HCO_3_^‒^-containing or HCO_3_^‒^-free buffer. Drugs were added either on the serosal (ser) and/or the mucosal (muc) side. Concentrations of the tested drugs were: bumetanide (10^-4^ mol·l^-1^ at the serosal side), methazolamide (10^-4^ mol·l^-1^ at the mucosal and the serosal side), SITS (10^-3^ mol·l^-1^ at the serosal or the mucosal side), DIDS (10^-3^ mol·l^-1^ at the serosal or the mucosal side), DNDS (5·10^-3^ mol·l^-1^ at the serosal or the mucosal side), and 1-EBIO (2·10^-3^ mol·l^-1^ at the serosal side). Values are given as difference of the maximal change in I_sc_ induced by the drug (peak) compared to the same baseline and are means ± SEM. * P < 0.05 (paired t-test).


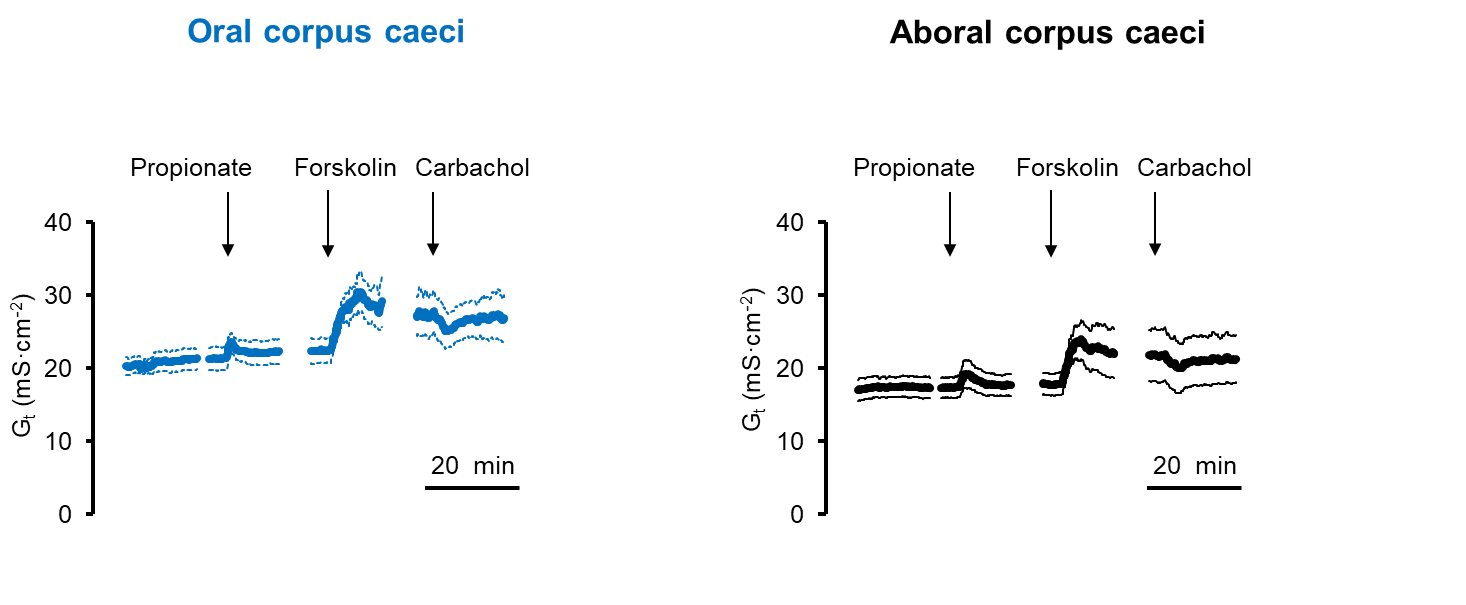


## **Fig. S1.** Response to Na propionate (2·10^-3^ mol·l^-1^ at the mucosal side) on tissue conductance (G_t_, mS·cm^-2^) in oral and aboral corpus caeci. Administration of propionate was followed by forskolin (5·­10^-6^ mol·l^-1^ at the mucosal and the serosal side) and carbachol (5·­10^-5^ mol·l^-1^ at the serosal side). Data are means (thick lines) ­± SEM (thin lines), n = 7. Line interruptions are caused by omission of time intervals in order to synchronize the tracings of individual records to the administration of drugs. The corresponding I_sc_ data of this series of experiments are depicted in Fig. 1A and B.
